# Serum S100B Protein is Specifically Related to White Matter Changes in Schizophrenia

**DOI:** 10.3389/fncel.2016.00033

**Published:** 2016-03-08

**Authors:** Berko Milleit, Stefan Smesny, Matthias Rothermundt, Christoph Preul, Matthias L. Schroeter, Christof von Eiff, Gerald Ponath, Christine Milleit, Heinrich Sauer, Christian Gaser

**Affiliations:** ^1^Department of Psychiatry, Jena University HospitalJena, Germany; ^2^St. Joseph-KrankenhausDessau-Roßlau, Germany; ^3^Department of Psychiatry, University of MuensterMuenster, Germany; ^4^Department of Psychiatry, St. Rochus HospitalTelgte, Germany; ^5^Department of Neurology, Jena University HospitalJena, Germany; ^6^Max Planck Institute for Human Cognitive and Brain Sciences and Clinic for Cognitive NeurologyLeipzig, Germany; ^7^Institute of Medical Microbiology, University of MuensterMuenster, Germany; ^8^Department of Neurology, School of Medicine, Yale UniversityNew Haven, CT, USA; ^9^Department of Psychiatry, Sophien- und Hufeland-KlinikumWeimar, Germany

**Keywords:** schizophrenia, S100B, white matter, voxel based morphometry, VBM, first episode psychosis

## Abstract

**Background:** Schizophrenia can be conceptualized as a form of dysconnectivity between brain regions.To investigate the neurobiological foundation of dysconnectivity, one approach is to analyze white matter structures, such as the pathology of fiber tracks. S100B is considered a marker protein for glial cells, in particular oligodendrocytes and astroglia, that passes the blood brain barrier and is detectable in peripheral blood. Earlier Studies have consistently reported increased S100B levels in schizophrenia. In this study, we aim to investigate associations between S100B and structural white matter abnormalities.

**Methods:** We analyzed data of 17 unmedicated schizophrenic patients (first and recurrent episode) and 22 controls. We used voxel based morphometry (VBM) to detect group differences of white matter structures as obtained from T1-weighted MR-images and considered S100B serum levels as a regressor in an age-corrected interaction analysis.

**Results:** S100B was increased in both patient subgroups. Using VBM, we found clusters indicating significant differences of the association between S100B concentration and white matter. Involved anatomical structures are the posterior cingulate bundle and temporal white matter structures assigned to the superior longitudinal fasciculus.

**Conclusions:** S100B-associated alterations of white matter are shown to be existent already at time of first manifestation of psychosis and are distinct from findings in recurrent episode patients. This suggests involvement of S100B in an ongoing and dynamic process associated with structural brain changes in schizophrenia. However, it remains elusive whether increased S100B serum concentrations in psychotic patients represent a protective response to a continuous pathogenic process or if elevated S100B levels are actively involved in promoting structural brain damage.

## Introduction

Structural abnormalities in the brains of schizophrenic patients have been frequently reported using post mortem (Harrison, 1999) and *in vivo* magnetic resonance imaging (MRI) techniques (Pearlson and Marsh, 1999; Wright et al., 2000; Gupta et al., 2015). Schizophrenia has been shown to be associated with ventricular enlargement and slightly decreased overall brain volume. Regional volume abnormalities were mainly localized in limbic structures and the temporal lobe (Bogerts et al., 1990; Wright et al., 2000). Neuroanatomical findings include decreased presynaptic and dendritic markers consistent with reduced neuron size and increased neuron density (Pakkenberg, 1993; Harrison, 1999). These findings and results from neuropsychological and neuroimaging studies of function have led to the notion of a disturbance of connectivity between brain regions (Andreasen et al., 1998; Lipska and Weinberger, 2002).

Consequently, abnormalities in interconnecting white matter (WM) structures have attracted interest (Davis et al., 2003). While investigations of global white matter volume have been inconclusive, local changes in white matter have been demonstrated in prefrontal cortex, temporo-parietal and parieto-occipital regions, splenium, cingulum, and posterior capsule, supporting the hypothesis of abnormal connectivity in schizophrenia (Davis et al., 2003; Kubicki et al., 2007; Ellison-Wright et al., 2008). However, the pathophysiological processes underlying structural abnormalities, their time dimension, and the relation to symptomatology and outcome are still to a large extent ambiguous. Structural abnormalities of the brain parenchyma like white matter myelin disturbance, deterioration of the neuropil, loss of synaptic connectivity, and functional impairment of oligodendrocytes have been proposed to contribute to the etiology of schizophrenia (Selemon and Goldman-Rakic, 1999; Davis et al., 2003; Katsel et al., 2011; Streitbuerger et al., 2012; Hercher et al., 2014).

S100B is a small acidic Ca^2+^-binding protein that is found in high abundance within the central nervous system (CNS). It is secreted by astrocytes and oligodendrocytes, and is also expressed in ependyma and neurons (Donato, 2001; Eldik and Wainwright, 2003; Schroeter et al., 2013). A post mortem histological study in human brain tissue showed that S100B immunostained cells in cortical regions have astrocytic morphology while most S100B positive cells located in white matter regions resembled oligodendrocytes (Steiner et al., 2007).

As a rather small protein with a molecular weight of 21kDa in its biologically active homodimeric form, S100B passes the blood brain barrier, and thus is detectable as a brain derived protein in peripheral blood (Reiber, 2001). Measurements of S100B in serum have been proven to valuably reflect the S100B concentration in cerebrospinal fluid (CSF) in healthy individuals as well as in patients with various neurological diseases (Reiber, 2001). In schizophrenic patients, increased S100B serum concentration is a repeatedly reported finding (Aleksovska et al., 2014).

Though many effects of S100B have been explored, the pathophysiologic role of elevated S100B levels in schizophrenia is not yet clarified. S100B is implicated in a number of Ca^2+^-dependent regulation processes, including phosphorylation, enzyme activity, cell metabolism, and signaling pathways (Donato, 2001; Rothermundt et al., 2004a). Extracellular effects of S100B depend on its local concentration. While nanomolar concentrations have been demonstrated to be neuroprotective and neurotrophic, micromolar concentrations have been shown to have neurotoxic effects and to induce apoptosis (Eldik and Wainwright, 2003; Gonçalves et al., 2008). Hence, S100B elevation can be considered to be a secondary attempt to protect nervous tissue, e.g., against glutamate induced stress (Ahlemeyer et al., 2000). Contrariwise, pathologic overproduction of S100B could also induce neuroinflammatory responses and thus make S100B a brain damaging agent itself (Eldik and Wainwright, 2003).

This study aims to ascertain whether there are associations between S100B elevation as a possible marker for glial function or dysfunction and local structural white matter changes in brains of schizophrenic patients. We utilized a voxel based morphometry (VBM) approach, since it is an established automated and thereby user-independent method well suited to detect local structural differences in the whole brain without prior definition of regions of interest by analysing *in vivo* magnetic resonance images (MRI; Ashburner and Friston, 2000; Honea et al., 2005). Building on prior knowledge from previous neuroimaging studies, interaction analyses, and histological post mortem studies, we hypothesized that an interaction between S100B and WM can be found in interconnecting fronto-temporal white matter structures (Steiner et al., 2008; Streitbuerger et al., 2012; Schroeter et al., 2013) and other repeatedly reported white matter structures within the frontal and temporal lobe as well as in cingulum bundle and corpus callosum (White et al., 2008).

## Methods

### Subjects

We investigated 17 consecutively admitted patients from the inpatient unit of the Department of Psychiatry of the Jena University Hospital fulfilling DSM IV criteria for schizophrenia (all Caucasians, epidemiologic data in Table 1). Eleven patients suffered their first acute psychotic episode (FEP), of which 8 were drug-naïve and 3 free of neuroleptic medication for at least 5 days. Six patients had been psychotic for more than one time, thus suffering from a recurrent acute psychotic episode (REP). Of those, 2 patients were still naïve in terms of neuroleptic drugs; 4 were free of neuroleptic medication for at least 4 days. Diagnosis was made for each patient by two independent board-certified psychiatrists (St. S., H. S.) and confirmed by structured clinical interview. Patients were compared to 22 healthy volunteers (all Caucasian, epidemiological data given in Table 1). None of the healthy controls had a personal or family history of psychiatric disorder. Subjects with any acute or chronic inflammatory disease or recent treatment with non-steroidal or steroidal anti-inflammatory drugs were excluded from the study. For all subjects, blood samples were taken for S100B serum level analysis and high-resolution MRI was performed. The study was approved by the Research Ethics Committee of the Jena University Hospital. All subjects gave written informed consent to participate in the study.

**Table 1:** **Epidemiological data and status of medication for each group**.

	**Control Group**	**First episode patients**	**Recurrent episode patients**
Number (N)	22	11	6
Gender (male/female)	9/13	5/6	1/5
Age (years ± SD)	34.73 (±10.30)	30.53 (±9.95)	42.00 (±9.28)
Drug-naïve (number)		8	2
Drug-free (number, duration of drug-free period)		3 (5–14 days)	4 (4 days–5 months)
Previous medication		Risperidone	Risperidone, Olanzapine, Haloperidole

### Acquiring and storage of blood samples

Venous blood (10 ml) was taken from an antecubital vein using a 19-gauge butterfly attached to a dry plastic syringe. Blood was allowed to clot for 30 min at room temperature. Serum was separated by centrifugation (10 min at 3000 rpm) within 2 h after collection and stored in 1 ml aliquots at −72°C. Mean duration of storage was 28.6 days (range: 12–56 days, SD: ±10.0 days). After thawing, sera were re-centrifuged and stirred carefully in order to avoid any inhomogeneities of the specimen.

### Analysis of S100B protein levels

S100B concentrations were determined by applying the LIAISON Sangtec 100 assay (AB Sangtec Medical, Bromma, Sweden), a quantitative automated luminometric immunoassay, according to the manufacturer's instructions. The assay's lower detection limit for S100B is 0.02 μgl^−1^. The intra-assay (within-run) imprecision (CVs) is between 2.6 and 6.4 %, depending on concentration. The inter-assay variation (CVs) is between 2.2 and 10.7 %. Analytical recovery ranges between 91 and 100.

### Acquisition of structural data and image processing

High-resolution MRI was performed on a 1.5 T Philips Gyroscan ACSII system. We acquired 256 sagittal slices using a T1-weighted sequence (TR = 13 ms, TE = 5 ms, flip angle 25°) with isotropic voxel size of 1 × 1 × 1 mm^3^. Data preprocessing and analysis was performed using SPM2 software (Statistical Parametric Mapping; Wellcome Department of Cognitive Neurology, London, UK).

For morphometric analysis of the data we used voxel-based morphometry (VBM). VBM is a fully automatic technique to computationally analyze of differences in local gray or white matter volume. This method involves the following steps: (i) spatial normalization of all images to a standardized anatomical space by removing differences in overall size, position, and global shape; (ii) extraction of gray and white matter from the normalized images; and (iii) analysis of differences in local gray and white matter volume across the whole brain (Ashburner and Friston, 2000). We applied an optimized method of VBM (Ashburner and Friston, 2000; Good et al., 2001) using the VBM2 Toolbox (http://dbm.neuro.uni-jena.de/vbm). The spatial normalization to the standard anatomical space was performed in a two-stage process. In the first step, we registered each image to the International Consortium for Brain Mapping (ICBM) template (Montreal Neurological Institute, Montreal, Canada), which approximates Talairach space. The normalized images of all subjects were averaged and smoothed with a 8 mm full-width at half-maximum (FWHM) Gaussian kernel; this averaged image was then used as a new template to reduce scanner- and population-specific bias. In the second normalization step, we locally deformed each image of our entire group to the new template using a non-linear spatial transformation. This accounts for the remaining shape differences between the images and the template and improves the overlap of corresponding anatomical structures. Finally, normalized images were corrected for non-uniformities in signal intensity and partitioned into gray and white matter, cerebrospinal fluid, and background using a modified mixture model cluster analysis. The resulting maps represent the local probability of belonging to a particular tissue type via voxel-wise values between 0 and 1. Because we applied spatial registration the same voxel location in each image should approximately correspond to the same brain structure. Using the probability values, we examined the relative concentration of one tissue type [i.e., the proportion of gray matter (GM) to other tissue types within a region]. We restricted the statistical analysis to areas with a minimum probability value of 0.1 to avoid possible edge effects around tissue borders. To remove unconnected non-brain voxels (e.g., rims between brain surface and meninges), we applied a series of morphological erosions and dilations to the segmented images (Good et al., 2001). Because these segmentations are often affected by noise we introduced spatial constraints based on neighboring voxels by using a Markov Random Field Model (Cuadra et al., 2005). The resulting white matter images were smoothed with a 12 mm FWHM Gaussian kernel.

### Statistical analysis

Statistical analysis of S100B serum levels was performed using the software package SPSS 15 for Windows. For evaluation of group differences, univariate analysis of variance (ANOVA) was performed using S100B concentration as the dependent variable, GROUP as a between-subject factor, and GENDER and AGE as covariates. To further investigate group differences found to be significant in ANOVA, we performed multiple pairwise group comparisons using a two-tailed Student's *t*-test with significance defined as *p* < 0.05 (corresponding to *p* < 0.0167 Bonferroni adjusted alpha).

Groupwise comparison of white matter imaging data was performed using a general linear model implemented in the software package SPM2. To account for variance related to age effects (due to different mean age between the patient groups), we included age as a confounding variable into the model. For resulting statistics, we set the significance threshold at *p* < 0.001. Only clusters exceeding the expected number of voxels per cluster (according to the Gaussian Random Field theory) were considered.

Assessment of associations between S100B concentration and structural abnormalities was realized using a general linear model in SPM2 with S100B concentration defined as a regressor. To account for age-related effects, we included age as confounding variable into the model. For pairwise group comparison, this type of analysis equals an interaction model (Smesny et al., 2010) testing for different regression slopes of white matter density related to S100B concentration between the groups in each voxel (S100B concentration vs. group interaction). Because variance was expected to differ between samples, we applied a non-sphericity correction. Again, all statistical images were thresholded at *p* < 0.001 and only clusters exceeding the expected number of voxels per cluster were reported. To support the anatomical labeling of our findings, we used the Mori MRI Atlas of Human White Matter (Mori et al., 2005).

## Results

### Sample characteristics and S100B serum concentration

**Figure 1:**
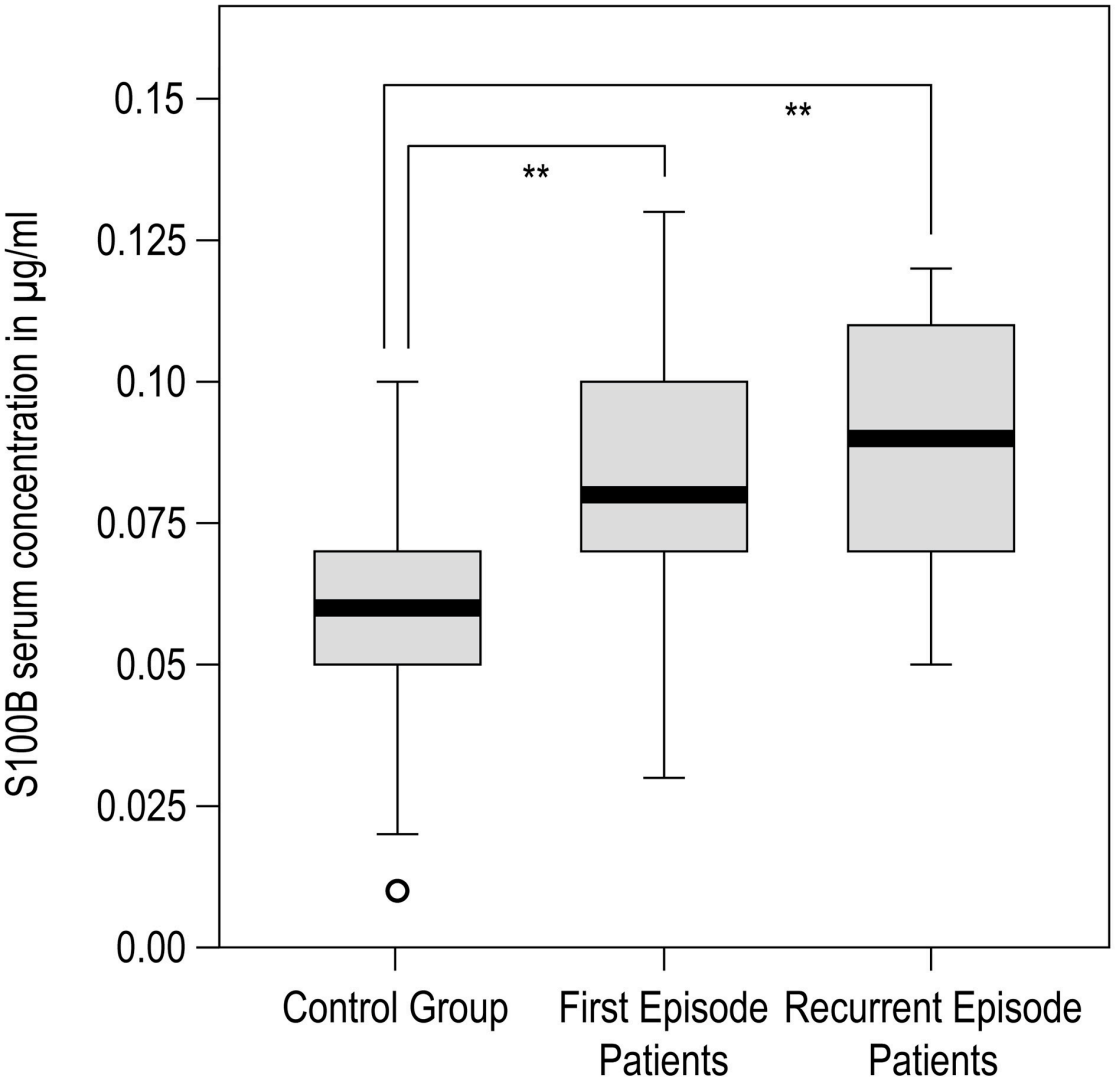
**SPSS generated boxplot for results of S100B serum concentration analysis**. Black bar within box: median, gray box: 25–75% quartiles; small black bars: minimum to maximum; circles: outliners. Significant group differences are marked with ^**^p < 0.01.

There was a difference in mean age between groups (Table 1). Group differences in mean age at the time of investigation originate from the natural age of onset and the course of the disease, leading to a consistently higher age in recurrent-episode patients (*t*-test: for FEP vs. REP: *p* = 0.001, for C vs. REP: *p* = 0.006, for C vs. FEP: *p* = 0.271). We took this into account by considering age as a covariate in all following analyses, thus assuring our findings are not due to effects of age. In terms of gender distribution, Fisher's exact test (gender by group) yielded *p* = 0.280 for C vs. FEP, *p* = 0.059 for C vs. REP, and *p* = 0.055 for FEP vs. REP, indicating that the gender distribution was not statistically significant. Also, univariate ANOVA and correlation analysis did not reveal any significant effect of GENDER or AGE on S100B serum concentration. Univariate ANOVA resulted in significant effects of GROUP on S100B levels (*F* = 6.310*, p* < 0.005; AGE and GENDER corrected model: *F* = 4.011*, p* = 0.009). The *t*-test results were congruent with previous findings that reported significantly higher S100B serum concentrations in both patient groups compared to controls (C vs. FEP: *p* = 0.006, C vs. REP: *p* = 0.008). No significant group differences were found between first and recurrent episode patients. Results and statistical information of S100B serum concentration analysis are presented in Table 2. A graphical presentation of the data is shown in Figure 1.

**Table 2:** **Results and corresponding statistical information of S100B serum concentration analysis, specified in μg/ml**.

	**Control Group**	**First episode patients**	**Recurrent episode patients**
Range	0.01−0.10	0.03−0.13	0.05−0.12
Mean ± SD	0.057 ± 0.023	0.084 ± 0.028	0.089 ± 0.026
Median	0.06	0.08	0.09
95% confidence interval	0.047−0.067	0.065−0.103	0.061−0.116

### VBM-based pairwise group comparison of brain imaging data

By performing a VBM-Based group comparison of MRI data, we found local white matter structural differences between patients and controls as well as between both patient groups. A detailed listing of the results of this analysis is presented in Table 3.

**Table 3:** **Results of group comparison of white matter brain structure between controls and patient groups (VBM-based analysis)**.

**Group comparison**	**Cluster size**	***T*-value (voxel)**	**Anatomical region**	**Assigned white matter structure**
C > FEP	251	4.25	Frontal lobe, medial orbital gyrus R	
C < FEP	209	4.37	Parietal lobe, region of gyrus Andrei Retzii L	Subcallosal white matter, Posterior cingulum bundle
C > REP	3606^*^	7.08	Occipital lobe, median area L	Inferior fronto-occipital fasciculus
	615	4.77	Cuneus L/R	
	194	4.03	Frontal lateral white matter compartment R	Corona radiata, Corticothalamic tract
	519	3.49	Frontal lobe, F1 region L	
C < REP	299	4.00	Temporal lobe, superior temporal gyrus, Angular gyrus R	Inferior longitudinal fasciculus, Arcuate fasciculus
	711	3.97	Cerebellum L	
FEP > REP	1033	5.40	Occiptal lobe, median and basal parts L	Inferior fronto-occipital fasciculus
	2064	4.82	Middle and upper pontine area, lateral circumference, cerebellar peduncle L	Corticospinal tract
	209	4.57	Parahippocampal area T4/T5 L	Fornix
	519	4.04	Middle and upper pontine area, lateral circumference, cerebellar peduncle R	Corticospinal tract
	265	3.78	Frontal lobe, F1 region, middle part R	
FEP < REP	n.s.	n.s.	n.s.	n.s.

*Between-group differences in white matter structure were found in reported clusters. Only clusters showing group differences at a significance level of p < 0.001 and exceeding the expected number of voxels per cluster according to the Gaussian Random Field theory are reported*.

* *p < 0.001 (cluster-level, FWE corrected for multiple comparisons). Anatomic labeling, corresponding cluster size and T-value are shown for each cluster. The type of group comparison is indicated in the form A < / > B, where A > B indicates lower white matter in group B compared to A and vice versa. Lateralization is marked with L for left and R for right hemisphere. Anatomical names of white matter structures were assigned using Mori: MRI Atlas of Human White Matter (Mori et al., 2005). C, control; FEP, first episode patients; REP, recurrent episode patients. C, control; FEP, first episode patients; REP, recurrent episode patients*.

When comparing healthy controls with patients suffering from their first psychotic episode (C vs. FEP), structural differences in white matter were located in the right medial orbital gyrus of the frontal lobe (diminished white matter in patients) and in white matter of the parietal lobe corresponding to the posterior cingulum bundle (increased white matter in patients). The VBM-based comparison of white matter between healthy controls and patients suffering from a recurrent psychotic episode (C vs. REP) yielded more and larger clusters denoting differences between these groups than between controls and first episode patients. The largest cluster indicating diminished white matter in recurrent episode patients was located in the medial area of the occipital lobe and was assigned to the inferior fronto-occipital fasciculus. Others were located in the cuneus, left frontal lobe, and frontal white matter assigned to the corticothalamic tract. Increased white matter in recurrent episode patients was found in the right superior temporal gyrus assigned to the inferior longitudinal fasciculus, and in the left cerebellum.

There were clusters indicating diminished white matter in first episode patients as compared to recurrent episode patients (FEP vs. REP). These were located in the left and right pontine area assigned to the corticospinal tract, the left occipital lobe, the left parahippocampal area, and the right frontal lobe.

### Associations of S100B serum levels and white matter structure in healthy controls

We did not find statistically significant associations between S100B serum concentration and white matter brain structure in healthy controls.

### VBM-based pairwise group comparison of associations between S100B serum levels and white matter brain structure

**Figure 2:**
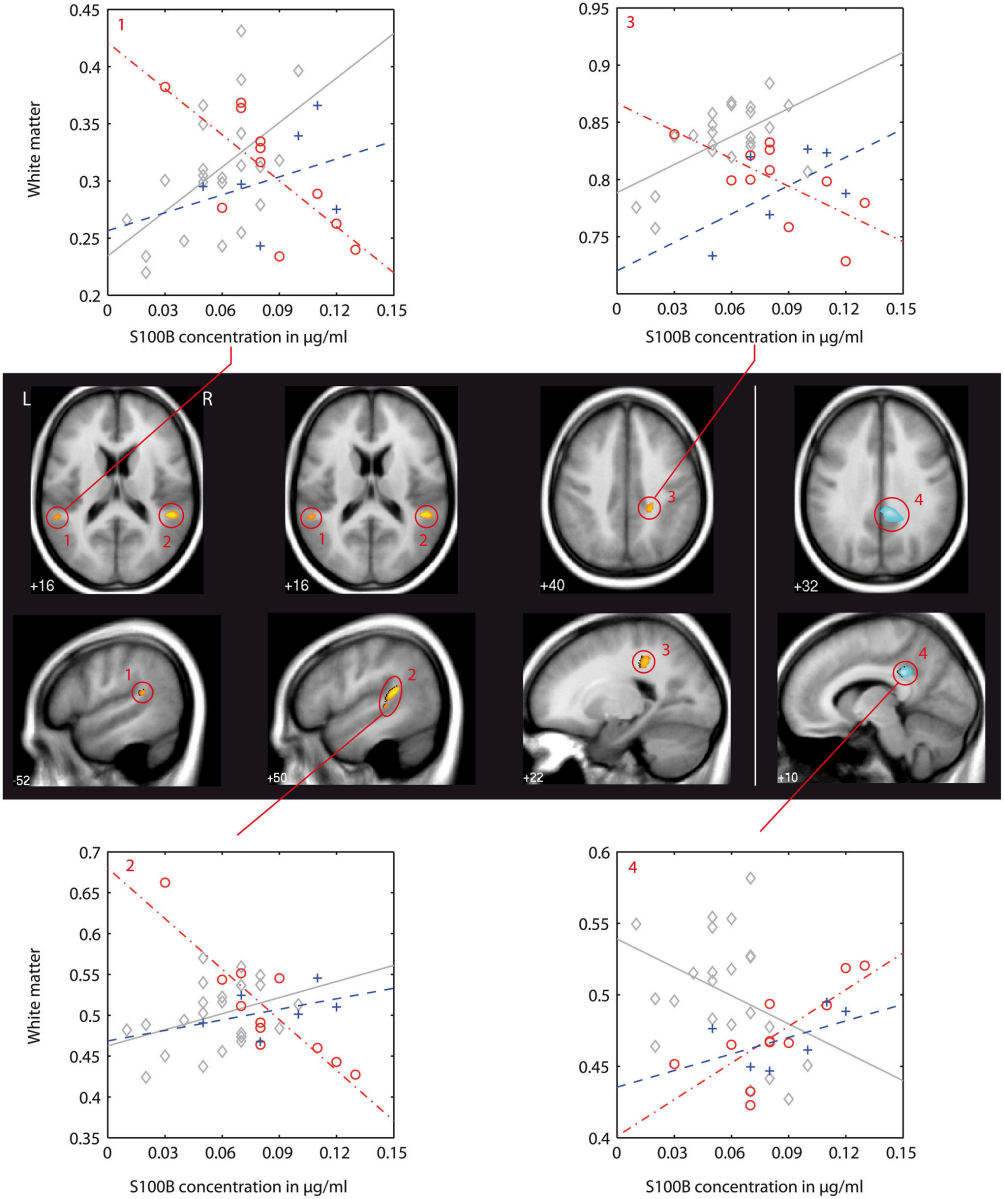
**VBM-based pairwise group comparison of associations between S100B concentration and white matter brain structure (interaction analysis): controls (C) vs. first episode patients (FEP)**. Clusters shown are significant as stated and numbered in Table 4 and are shown as overlay to the T1 average image in axial and sagittal plane for each group comparison as indicated below anatomical images. The T1 averaged image was obtained from images of all participants of this study. Positive interaction (regression of group *A* > *B*) is indicated by red/yellow color, negative interaction (*A* < *B*) by blue color (colorbar with corresponding *T*-values shown in Figure 3). Numbering of clusters is as according to Table 4. Scatter plots showing S100B concentration against white matter values (obtained from 1st Eigenvariate of its corresponding cluster) and corresponding regression lines are demonstrated exemplarily for each found interaction pattern. Color coding in scatter plots: gray, C, controls; red, FEP, first episode patients; blue, REP, recurrent episode patients.

**Figure 3:**
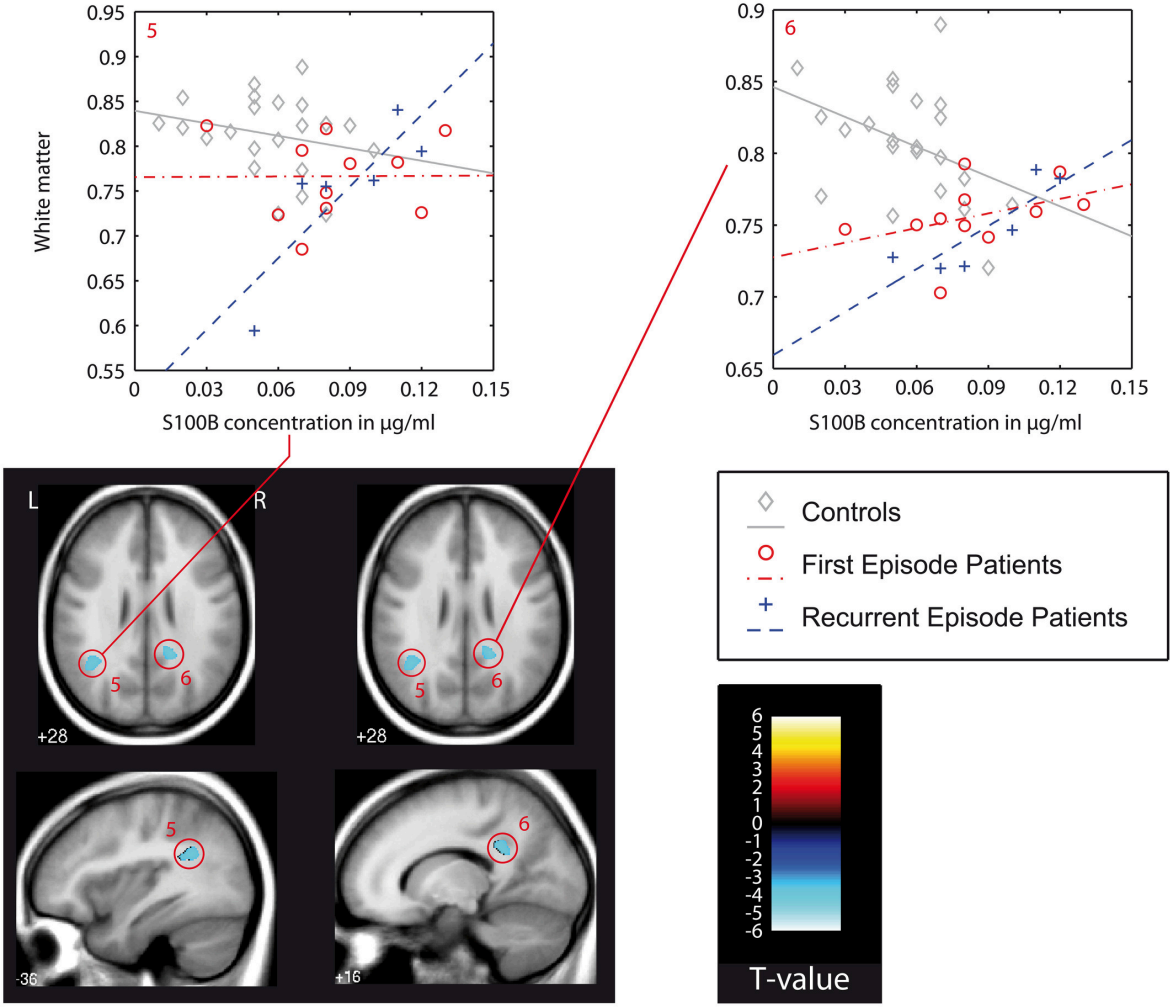
**VBM-based interaction analysis: control group (C) vs. recurrent episode patients (REP)**. Presentation as in Figure 2. In this figure also: color bar (*T*-values) and color/symbol coding for scatter plots in Figures 2–4: gray, C, controls; red, FEP, first episode patients; blue, REP, recurrent episode patients.

**Figure 4:**
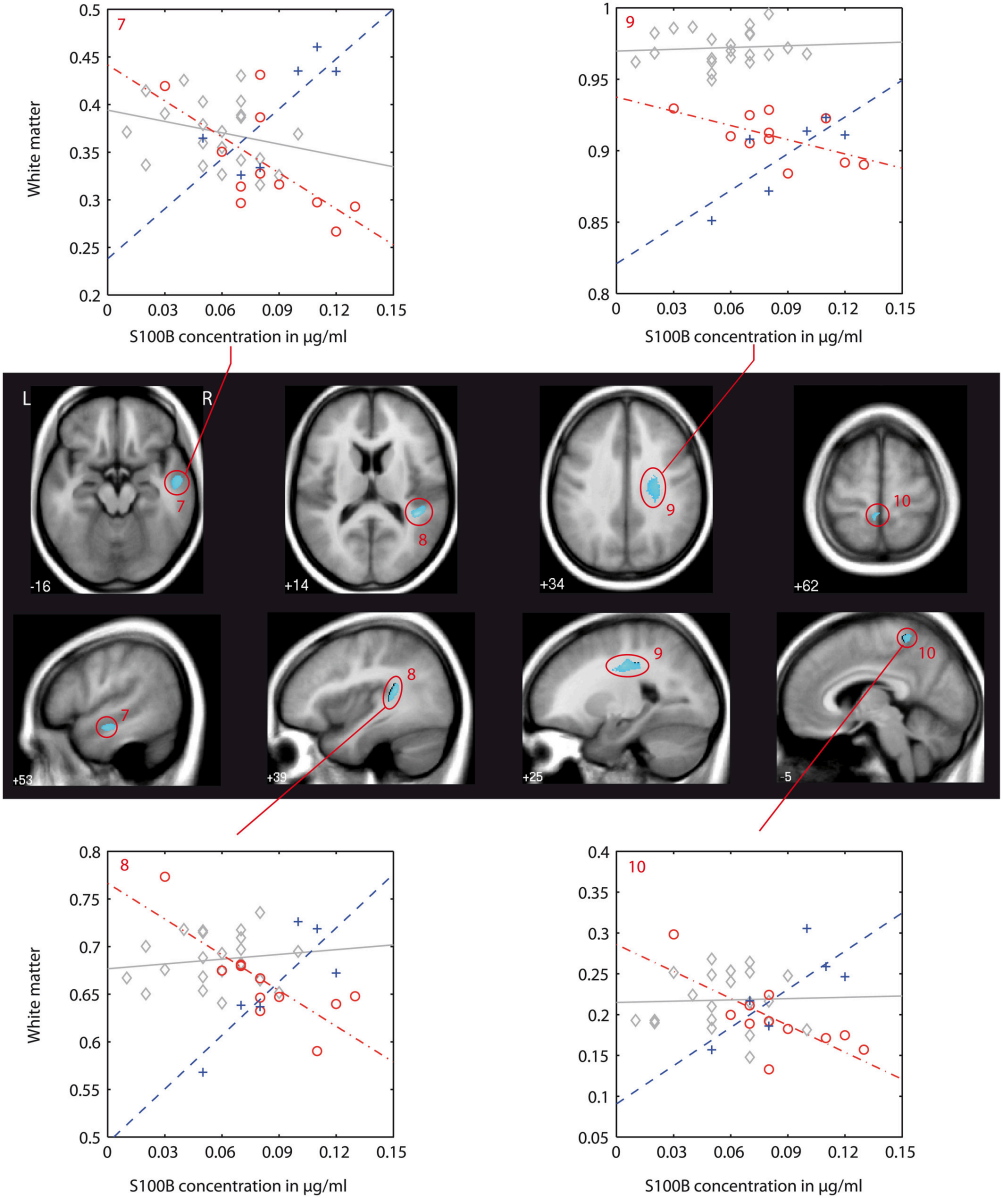
**VBM-based interaction analysis: first episode patients (FEP) vs. recurrent episode patients (REP)**. Presentation as in Figure 2. Color coding in scatter plots: gray, C, controls; red, FEP, first episode patients; blue, REP, recurrent episode patients.

We found differences in the association between local white matter structures and S100B concentration (denoted as *interaction* in the following text) between patients and controls as well as between both patient groups. A detailed listing of the results of this VBM-Based interaction analysis is presented in Table 4. Figures 2–**4** show localization and dimensions of each significant cluster listed in Table 4 as an overlay onto the averaged T1-image of all subjects in axial and sagittal plane. Also shown are scatter plots and corresponding regression lines of S100B concentration vs. white matter values obtained from the 1st eigenvariate of the corresponding cluster.

**Table 4:** **Results of pairwise group comparison of associations between S100B concentrations and white matter brain structure (VBM-based interaction analysis)**.

**Interaction**	**Cluster Number**	**Cluster Size**	***T*-Value (voxel)**	**Anatomical region**	**Assigned white matter structure**
C > FEP	1	175	3.80	Temporal lobe, superior temporal gyrus L	Inferior longitudinal fasciculus Arcuate fasciculus
	2	880	5.13	Temporal lobe, superior temporal gyrus R	Inferior longitudinal fasciculus Arcuate fasciculus
	3	400	4.17	Dorsal parietal white matter compartment Postcentral region R	Superior longitudinal fasciculus
C < FEP	4	1214	5.55	Parietal white matter compartment Postcentral region R	Posterior cingulum bundle
C > REP	n.s.	n.s.	n.s.	n.s.	
C < REP	5	818	4.28	Temporal lobe, superior temporal gyrus Angular gyrus L	Superior longitudinal fasciculus Arcuate fasciculus
	6	617	4.58	Parietal white matter compartment Postcentral region R	Posterior cingulum bundle
FEP > REP	n.s.	n.s.	n.s.	n.s.	
FEP < REP	7	416	4.19	Temporal lobe, superior temporal gyrus, Insula R	Inferior longitudinal fasciculus
	8	945	5.32	Temporal lobe, T1/T2 region	Arcuate fasciculus
	9	1289	4.84	Fronto-parietal white matter compartment R	Superior longitudinal fasciculus
	10	220	4.06	Parietal lobe, Postcentral region	

Abbreviations, labeling and data structure as in Table 3. Interaction type is indicated in the form A < / > B, whereby A > B indicates a significantly steeper gradient of regression (association between S100B and white matter) of A as compared to B and vice versa. Clusters are numbered for easier identification in figures and discussion. C, control; FEP, first episode patients; REP, recurrent episode patients.

Differences in the association between S100B and white matter between healthy controls and first episode patients (C vs. FEP) could be found in the left and right superior temporal gyrus assigned to the inferior longitudinal fasciculus (negative association in patients, illustrated in Figure 2: clusters 1, 2, and 3) and in the right postcentral dorsal white matter compartment assigned to the posterior cingulum bundle (steeper regression gradient in patients, Figure 2: cluster 4). In both cases, local white matter was diminished in patients.

In recurrent episode patients, there was a similar finding in the right postcentral white matter (posterior cingulum bundle, Figure 3: cluster 6). Also, there was a finding located in the superior temporal gyrus of the left temporal lobe (Figure 3: cluster 6), but the association between S100B and white matter was positive in recurrent episode patients and negative in first episode patients.

This type of interaction (negative association between S100B and white matter in first episode patients, positive association in recurrent episode patients) was also found in the direct comparison of both patient groups. With the chosen level of significance, a cluster in the right superior temporal gyrus could be identified (Figure 3: cluster 7). Additionally, a larger cluster (Figure 3: cluster 9, cluster-size 1289) could be detected in the right fronto-parietal white matter compartment, and was assigned to the superior longitudinal fasciculus. Other findings were located in the right temporal lobe and left postcentral region.

### VBM-based pairwise group comparison between S100B and gray matter

We followed a hypothesis driven approach and considered S100B as a protein associated with white matter (Schroeter et al., 2013). However, to complete the picture, we additionally calculated results for gray matter in the same way as we did for white matter. It resulted in only 2 small clusters in the bordering region between gray and white matter—one next to the posterior cingulum bundle and one in the temporal lobe approximately corresponding to the neighborhood of the localization of cluster 2 in the white matter analysis but with much smaller extent (data not shown).

## Discussion

This interaction analysis utilized a combined approach of voxel based brain morphometry and quantitative analysis of S100B serum concentration. It aimed to identify correlations between S100B concentration and white matter brain structure changes for schizophrenic patient groups at different stages of the disorder as compared to healthy controls. It also intended to show anatomic localizations of group differences. To minimize possible confounding effects, we ensured that all patients were free of antipsychotic medication at the time of investigation. Recurrent episode patients were significantly older at the time of investigation than controls and first episode patients. We took this into account by considering age as a nuisance variable in all VBM-based analyses and demonstrated that our findings were not due to age effects. Furthermore, investigation of patient subgroups (first episode and recurrent episode patients) allowed us to characterize patterns of S100B-associated structural brain abnormalities not only at the time of initial schizophrenic manifestation, but also at a recurrent episode stage.

### S100B serum concentration

S100B serum concentration was significantly increased in both patient groups when compared to healthy volunteers (Table 2, Figure 1), which constitutes a replication of findings in other first episode and chronic patient populations (Rothermundt et al., 2004a; Aleksovska et al., 2014). We did not find differences in S100B serum concentration between first and recurrent episode patients. When interpreting the latter result, one should bear in mind that, at the time of investigation, patients of both groups were unmedicated (or drug naïve) and had suffered an acute psychotic episode. Based on findings from other studies demonstrating that S100B decreases only in some patients when medicated (Rothermundt et al., 2001), or that it stays elevated even after 24 weeks of treatment in chronic patients (Rothermundt et al., 2004b), it is reasonable to assume that S100B elevation in schizophrenic patients is associated with pathophysiological processes inherent to psychotic episode in schizophrenia or, more likely, the disorder itself (Rothermundt et al., 2004a).

### VBM-based pairwise group comparison of brain imaging data

In this section, we will discuss the results of the pairwise group comparison of white matter brain imaging data as presented in Section *VBM-Based Pairwise Group Comparison of Brain Imaging Data* and Table 3. While the main goal of this study was to identify possible interrelations between structural white matter changes and S100B concentration (see Sections *Associations of S100B Serum Levels and White Matter Structure in Healthy Controls* and *VBM-based Pairwise Group Comparison of Correlations between S100B Serum Levels and White Matter Brain Structure*), presentation and discussion of just the structural findings may be helpful for understanding and embedding results of the following interaction analysis into a broader neuroanatomical and pathophysiological context.

#### First episode patients

We found differences in local white matter structures already present in the brains of first episode patients when compared to controls, even though most of these patients were drug-naïve. These differences were located in frontal regions (diminished WM) and parietal white matter structures (elevated WM) corresponding to the posterior part of the cingulum bundle.

Only few studies analyzing whole brain white matter structure in untreated first episode patients have been conducted so far (Kubicki et al., 2007; Kyriakopoulos and Frangou, 2009; Samartzis et al., 2014). Reductions of fractional anisotropy (FA) were found, among other regions, in the right parietal WM (Kyriakopoulos and Frangou, 2009). A recent DTI-based study revealed lower FA bilaterally in regions corresponding to the superior and inferior longitudinal fasciculus, the forceps major, the thalamic radiation, and the corpus callosum (Pérez-Iglesias et al., 2010). These and our findings strengthen the notion of disturbed white matter integrity being present already in very early stages of the disorder (Pantelis et al., 2005; Begré and Koenig, 2008; Kyriakopoulos and Frangou, 2009).

#### Recurrent episode patients

In recurrent episode patients we found both, areas with diminished and increased local white matter (when compared to the control group). The largest cluster (3606 voxels) with the highest *T*-score (7.08) showing locally diminished WM was located in the left occipital lobe in the region corresponding to the inferior fronto-occipital fasciculus. Another area of diminished WM was located in the right frontal white matter corresponding to the corticothalamic tract. These results are in line with previous VBM-based studies (Suzuki et al., 2002), as well as DTI-based studies that describe abnormalities of those structures in terms of decreased FA or reduced track length (Kubicki et al., 2005; Mitelman et al., 2007; Ellison-Wright and Bullmore, 2009). Abnormalities of these WM structures provide evidence supporting the hypothesis of cerebral dysconnectivity in schizophrenia (Konrad and Winterer, 2008; Ellison-Wright and Bullmore, 2009).

Increased WM was found in the left cerebellum and the temporal white matter corresponding to the arcuate fasciculus, structures that have been repeatedly reported to be altered in schizophrenia as well (Douaud et al., 2007; Kubicki et al., 2007; Ellison-Wright and Bullmore, 2009). The possible meaning and relevance of temporal lobe findings are discussed in more detail in Section *VBM-based Pairwise Group Comparison of Correlations between S100B Serum Levels and White Matter Brain Structure*.

#### First episode vs. recurrent episode patients

When comparing the first episode and recurrent episode patient groups, we found clusters indicating locally diminished white matter in the REP group but not in the FEP group. Although this was not a longitudinal study and these results must be interpreted cautiously, these findings may refer to an ongoing process of structural remodeling during the disease course. Such a process has been proposed in several previously published studies (DeLisi, 1999; Pantelis et al., 2003; Brans et al., 2008; Pol and Kahn, 2008).

In our sample, we found that the fornix and the inferior fronto-occipital fasciculus (see Section *Recurrent Episode Patients*) differ significantly between the FEP and REP groups. The fornix is one of the main pathways connecting the hippocampus with other brain regions and has repeatedly been shown to be altered in schizophrenia (Kubicki et al., 2005; Kuroki et al., 2006). It has also been associated with memory impairment (Nestor et al., 2007). Also, we found that the cerebellar peduncles and other structures corresponding to the corticospinal tract differ significantly between the patient groups. Structural changes of the cerebellar peduncles could contribute to some cognitive symptoms, since they are part of the frontal-thalamic-cerebellar circuitry that is thought to be disturbed in schizophrenic patients (Andreasen et al., 1998). The idea of a possible change in these structures over the course of the disease is supported by results of some DTI-based studies which reported reduced FA in early (Kyriakopoulos et al., 2008) and later disease stages (Okugawa et al., 2006), but not in chronic schizophrenic patients (Wang et al., 2003).

### VBM-based pairwise group comparison of correlations between S100B serum levels and white matter brain structure

In the following, we will discuss the results of the VBM-based interaction analysis (for cluster numbering and anatomical localization, see Table 4 and Figures 2–4) for all three group comparisons made (C vs. FEP, C vs. REP, and FEP vs. REP). This type of analysis was intended to test which local differences in structural imaging data between groups can be explained by differences of the chosen regressor S100B.

In first episode patients, S100B-associated differences to controls were located in the white matter of regions of the left and right superior temporal gyrus (STG), and in the postcentral region (clusters 1, 2, and 3). At these localizations, increased S100B concentrations were associated with diminished local white matter values (negative correlation). In the right cingulum bundle (cluster 4), first episode patients showed a positive correlation between S100B concentrations and white matter values.

Structural abnormalities of the temporal lobe rank among the most consistently reported morphometric findings in first episode patients (Bogerts et al., 1990; Pearlson and Marsh, 1999; Honea et al., 2005). As extensively discussed elsewhere, volume differences might follow from changes in neuropil and white matter (Selemon and Goldman-Rakic, 1999; Davis et al., 2003; Walterfang et al., 2005). Furthermore, white matter volume differences involving the inferior longitudinal fasciculus have been found in a subgroup of schizophrenic patients (Sigmundsson et al., 2001). Results from studies investigating white matter fiber tracts with more subtle *in vivo* methods, such as diffusion tensor imaging (DTI) or magnetization transfer imaging (MTI), strengthen the hypothesis that disturbed integrity of white matter structures is present in patients suffering from schizophrenic psychosis (Kubicki et al., 2005, 2007; Ashtari et al., 2007). Therefore, differences in the superior temporal gyrus (STG) are of special interest, as these structures include Heschl's gyrus and planum temporale. Disturbances in these structures and the inferior longitudinal fasciculus connecting Broca's and Wernicke's area may account for the pathophysiology of hearing voices (Dierks et al., 1999; Ashtari et al., 2007).

Alterations of the cingulum bundle have been consistently reported in previous studies investigating white matter abnormalities in schizophrenia (Sigmundsson et al., 2001; Kubicki et al., 2003, 2005; Shergill et al., 2007; Peters et al., 2008). As the cingulum bundle connects limbic structures—and is also known to interconnect the thalamus, prefrontal, parietal, and temporal lobes with the cingulate gyrus—local pathology of these structures already detectable in first episode patients might contribute to the hypothesis of dysconnection of the hippocampal/temporolimbic complex with the dorsolateral prefrontal cortex (Lipska and Weinberger, 2002; Mori et al., 2005).

While there was a joint finding in the posterior cingulum bundle of both patient groups, the striking result of our analysis was significant differences between both patient groups. These are localized in the right superior temporal gyrus (superior and inferior longitudinal fasciculus), right middle temporal lobe, in the postcentral region, and in a large region of the right fronto-temporal white matter that corresponds to the right superior longitudinal fasciculus. In all of these regions, first episode patients had a negative correlation of S100B concentration to white matter values while recurrent episode patients had a positive correlation (Figure 4).

There is a significant body of evidence for disturbed white matter configuration in temporal regions, including the STG, the insula (Ashtari et al., 2007; Mitelman et al., 2007; Shergill et al., 2007; Ellison-Wright and Bullmore, 2009), and the fronto-temporal white matter structures including the superior longitudinal fasciculus (Kubicki et al., 2005; Samartzis et al., 2014). A progression of structural temporal changes has been reported (Kasai et al., 2003a,b; Ellison-Wright et al., 2008) while another follow-up study did not find significant progression of structural changes in temporal regions (DeLisi and Hoff, 2005).

Although based on a cross-sectional analysis, our results suggest differences in the structural abnormalities of these regions between schizophrenic patients of earlier (FEP) and later (REP) stages in terms of a diverging correlation with S100B concentrations.

### Summary and conclusions

There were significantly different correlations between S100B concentration and local white matter formations between both patient groups and healthy controls, as well as between first episode and recurrent episode patients. Structural white matter changes showing a different correlation with S100B between groups are located in brain regions that have been previously described extensively in the literature to be affected in schizophrenic patients. Also, these structures are integral parts in hypotheses about the physical foundation of neurocognitive symptoms in schizophrenia. A divergent association of S100B concentration with white matter structural changes could already be detected in the very early stage of the disorder (FEP). While there was a finding in the cingulum bundle that appears to be stable during the course of the disease, the correlation of S100B concentrations with white matter structural changes was found to be reversed between patient groups at different stages of disorder, especially in the white matter of the superior temporal gyrus corresponding to the inferior longitudinal fasciculus and fronto-temporal white matter corresponding to the superior longitudinal fasciculus.

We conclude that S100B is involved in an ongoing dynamic process associated with local structural changes in white brain matter of schizophrenic patients. Still, the question is open whether frequently found increased S100B serum concentration in psychotic patients at different stages of disorder is a secondary attempt of protection against an ongoing harmful process or if pathologically elevated S100B levels themselves can be held responsible for structural brain damage, either directly or as part of a biochemical network (Hanson and Gottesman, 2005; Monji et al., 2009).

Considering the range of measured S100B concentrations (Table 2), massive glial or neuronal destruction is unlikely to be the cause of S100B increase in schizophrenia (Rothermundt et al., 2004a).

A recent study by Streitbuerger et al. (2012) investigated the correlation between serum S100B levels and gray matter and white matter parameters with MRI (T1-weighted and diffusion tensor imaging) in healthy subjects. Here, S100B was specifically related to the diffusion tensor imaging parameters fractional anisotropy and radial diffusivity, the latest an indicator of myelin changes, in the corpus callosum, anterior forceps, and superior longitudinal fasciculus in female subjects. In contrast, there was no association between gray matter T1 data and S100B. Histological data confirmed a co-localization of S100B with oligodendrocyte markers in the human corpus callosum in this study. The authors showed additionally, that S100B was most abundantly expressed in the corpus callosum according to the whole genome Allen Human Brain Atlas. Based on these data, one might conclude that serum S100B represents a biomarker for white matter tracts and, consequently, oligodendroglia, which might have led to the association between white matter parameters and schizophrenia in our study, without relevant effects on gray matter.

Given the fact that S100B is involved in many different regulation processes and also linked to glutamatergic and cytokine systems (Ahlemeyer et al., 2000; Tramontina et al., 2006; Müller and Schwarz, 2007; Gonçalves et al., 2008), elevated S100B in schizophrenic psychosis could be an expression of other cross-linked and ongoing biochemical pathophysiological processes. Thus, the exact relation of S100B with structural changes remains open. On the other hand, S100B is a protein that exerts paracrine and autocrine effects on neurons and glia, and thus plays an important role in cell proliferation and differentiation, cellular energy metabolism, and cytoskeletal modification (Rothermundt et al., 2004a; Gonçalves et al., 2008). Hence, S100B might also be directly involved in processes leading to structural white matter changes in schizophrenia.

### Limitations of the study

We included patients at different stages of the disorder to cover the entire course of the disease in a cross-sectional design. Cross-sectional studies have their limitations in the interpretation of the results when discussing questions about progression of structural changes. The sample sizes used in this study are considered to be moderate (Honea et al., 2005). However, an essential aspect of this study was to investigate unmedicated patients and also to compare first episode patients with patients at a later stage of the disease. Thus, the sample size was limited due to the small number of patients fulfilling these criteria.

All patients participating in this study were either neuroleptic-naïve or unmedicated. Due to practical and ethical reasons, of those who were unmedicated, some patients were off medication only for 4 respective 5 days, and some individuals had received haloperidole as previous medication. Considering the effects of antipsychotic medication on brain structure (Lieberman et al., 2005), it would have been desirable to have had a longer medication-free period before obtaining MRI data, so we cannot completely rule out a possible effect of medication on the results, especially in the REP group.

Patient groups differed in mean age. To ensure that found differences between these groups were not predominantly caused by effects of age, we included age as nuisance variable in all analyses. By this method a possible influence of age on the results can be diminished but not completely be ruled out.

A correlation (*r* = 0.538, *p* < 0.001) between S100B and body mass index (BMI) has been reported in healthy controls (Steiner et al., 2010a), though such correlation was not found in patients suffering from schizophrenia (Steiner et al., 2010b). However, S100B might have other sources than the brain, e.g., adipose tissue (Gonçalves et al., 2010) and it is recommendable to consider body mass index in future studies.

There was also an imbalance in the gender ratio. Differences in brain structure between men and women are at least in part a matter of overall size. While such size and shape differences are controlled by normalizing each image to the ICBM template (see *Section Acquisition of Structural Data and Image Processing*), a possible influence of gender on our results cannot completely be ruled out. Also, gender specific correlations between serum S100B and white matter parameters have been found in a previous study (Streitbuerger et al., 2012). Hence, we re-performed analyses with a reduced sample including female subjects only (data not shown). Although this lead to a considerable reduction of sample size, main effects were comparable (findings in cingulum bundle, temporal lobe findings).

For the statistical analysis, a stringent threshold of *p* = 0.001 was chosen to exclude false positives resulting from unaccounted confounding variables (Ashburner and Friston, 2000). Furthermore, having exclusively found significant changes in anatomical structures previously described to be altered in schizophrenia in a non-region-of-interest-approach, it is reasonable to assume that our results are valid.

We used VBM to find local differences in white matter values of T1 weighted MR images and assigned detected clusters to anatomical fiber structures according to an atlas of human brain white matter, presuming that localizations of clusters found in our analysis correspond to these structures. This method cannot show the fiber tracts themselves, including their directions and disruptions. To prove that these fiber tracts are indeed disrupted and that the disruptions are associated with S100B, future studies in this field should also utilize techniques such as DTI or MTI.

## Author contributions

BM performed data analyses with SPM and SPSS, wrote MATLAB scripts to plot graphs, made tables and figures, and conceptualized and wrote the article. SS had the original idea and made the concept of this interaction analysis. He also screened patients, made diagnoses and obtained MRI-data. MR, GP, CV did the measurement of S100B protein, wrote the methods part of S100B measurement, contributed to and revised the text of the manuscript, especially in the discussion regarding S100B protein. CP assigned the found clusters to anatomical white matter structures, did proof reading, and contributed to the discussion regarding neuroanatomical points. MS made major contributions to introduction and discussion and especially helped with connecting the results to findings of other studies in this field. CM did additional statistical analysis, made extensive literature research on the topic, maintained the literature database, wrote most of the introduction, and contributed to the logical structure of the manuscript. HS is the head of department. He provided all means to conduct the study, contributed to the original design, diagnosis of patients, obtaining of MRI-data, and to the text of the manuscript. CG is the senior author. He provided all knowledge and means to perform VBM analysis, wrote the VBM toolbox and scripts to make slice overlays. He provided intensive help in preparing and performing computer analyses, and wrote all MR-related parts of the manuscript.

## Funding

Stefan Smesny was supported by the German Research Foundation (DFG), grant Sm 68/1-1. Christian Gaser was supported by the German Federal Ministry of Education and Research (BMBF), grant 01EV0709.

### Conflict of interest statement

MR received speaker's honoraria from the companies Janssen-Cilag and Servier. The other authors declare that this study was conducted in the absence of any commercial or financial relationships that could be construed as a potential conflict of interest. The reviewer KS and handling Editor declared their shared affiliation, and the handling Editor states, that the process nevertheless met the standards of a fair and objective review.
